# A novel porous media-based approach to outflow boundary resistances of 1D arterial blood flow models

**DOI:** 10.1007/s10237-019-01122-8

**Published:** 2019-03-21

**Authors:** Alberto Coccarelli, Arul Prakash, Perumal Nithiarasu

**Affiliations:** 1 Zienkiewicz Centre for Computational Engineering, College of Engineering, Swansea University, Swansea, UK; 2 Department of Applied Mechanics, Indian Institute of Technology Madras, Chennai, India; 3 VAJRA, Indian Institute of Technology Madras, Chennai, India

**Keywords:** Arterial blood flow, Terminal resistance, Poro-elastic model, Systemic circulation, Microcirculation

## Abstract

In this paper we introduce a novel method for prescribing terminal boundary conditions in one-dimensional arterial flow networks. This is carried out by coupling the terminal arterial vessel with a poro-elastic tube, representing the flow resistance offered by microcirculation. The performance of the proposed porous media-based model has been investigated through several different numerical examples. First, we investigate model parameters that have a profound influence on the flow and pressure distributions of the system. The simulation results have been compared against the waveforms generated by three elements (RCR) Windkessel model. The proposed model is also integrated into a realistic arterial tree, and the results obtained have been compared against experimental data at different locations of the network. The accuracy and simplicity of the proposed model demonstrates that it can be an excellent alternative for the existing models.

## Introduction

One-dimensional flow modelling in cardiovascular systems has gained significant popularity in the recent years due to its potential applications in different areas such as fundamental understanding of blood flow (Mynard and Nithiarasu 2008), fractional flow reserve (FFR) calculations (Boileau et al. 2017), aneurysm detection (Sazonov et al. 2017), hypertension (Segers et al. 2007) and many other applications (Coccarelli and Nithiarasu 2015; Coccarelli et al. 2016, 2017, 2018). In many of these applications, prescribing accurate boundary conditions is essential to obtain sensible results. The material properties and geometry are the other important aspects that influence results. Among the boundary conditions, resistance boundary conditions at the extremities play an extremely important role in determining the accuracy of predictions. In the following paragraphs, the most commonly used methods for prescribing resistance at the extremities of a systemic circulation model are reviewed.

The easiest way for modelling vascular bed effects is to employ a pure resistive model. By adopting this approach, the downstream vasculature is simply seen as a pressure drop, and thus flow and pressure waveforms at the outflow node are in phase. Although this model has been largely used in several works (Schaaf and Abbrecht 1972; Avolio 1980; Wan et al. 2002; Sherwin et al. 2003a, b; Matthys et al. 2007), the effect of downstream vascular compliance is not accounted for, and this may lead to poor results. More advanced models for the treatment of the outflow boundary conditions include the structured tree approach (Olufsen 1999; Olufsen et al. 2000) and its derived versions (Brown 1996; Cousins and Gremaud 2012, 2014), tapering vessels (Mynard and Nithiarasu 2008; Low et al. 2012; Hasan et al. 2018) and the three elements (RCR) Windkessel model (Porenta et al. 1986; Stergiopulos et al. 1992; Alastruey 2006; Urquiza et al. 2006; Formaggia et al. 2006; Du et al. 2015).

The latter model undisputedly represents the most popular approach for modelling the downstream vasculature effects, and it was employed in the most advanced arterial circulation frameworks (Reymond et al. 2009; Alastruey et al. 2014; Blanco et al. 2014, 2015; Mynard and Valen-Sendstad 2015; Carson and Van Loon 2017). It consists of two resistances and one capacitor, with the latter representing arterial compliance. Alastruey et al. (2008) have increased the robustness of the RCR Windkessel model by including time-dependent resistances in order to simulate control mechanisms in the cerebral circulation. It is important to mention that other models, like (Olufsen 1999; Cousins and Gremaud 2012, 2014), allow the integration of characteristic regulatory mechanisms of microcirculation.

As outlined by Olufsen (1999), pure resistive and Windkessel models are lumped and thus wave propagation effects are not included in the part of arterial system they represent. Furthermore, using this latter model for outflow conditions may lead to nonrealistic phase lag between flow and pressure because of artificial reflections (Olufsen 1999; Vignon-Clementel and Taylor 2004). Structured arterial tree models are able to tackle this issue, but they involve higher computational costs for treating the boundaries (Guan et al. 2016). Another disadvantage related to the use of Windkessel models is that the model parameters strongly depend on the vascular bed represented. In accurate representations of arterial tree, like (Blanco et al. 2015; Mynard and Valen-Sendstad 2015), the specific resistance coefficients for each terminal RCR model have been determined by considering the amount of blood flow reaching that region. Compliance parameters of the downstream circulation are generally calculated by assuming proportional distributions with respect to either terminal resistances or compliances. By having optimal values for the total resistance and compliance, the overall wave shape can be well approximated; however, these quantities are not easy to measure and the pulse profiles are extremely sensitive to these parameters. In addition to this, if there is a need to either extend or reduce the network, new RCR model coefficients need to be determined for representing the new downstream system. Similarly, tapering vessels approach is limited by the fact that tapers are artificial and therefore their length must be tuned depending on which fraction of the vascular bed branching they represent. The porous media model proposed addresses some of the issues identified and provides a natural extension of the arterial flow network at the boundaries.

The porous media models have been already used for describing arterial blood flow and perfusion within soft tissues, see for example Khaled and Vafai (2003), Khanafer and Vafai (2006) and Iasiello et al. (2014). However, none of these models have been adopted for studying blood flow in large network problems, such as the systemic circulation. Therefore, it is clear that progress can still be made in the development of more robust and physically based methodologies. In the present work a novel, physically motivated method for treating outflow boundary conditions in arterial networks is introduced. The idea behind this work is based on the proposition that the downstream resistance of a blood flow network can be assimilated to an elastic porous media. The new methodology proposed is presented in Sect. 2. This includes the model derivation and the solution procedure adopted throughout the study. In Sect. 3 the model is tested on several different problems, including a flow network. Some important conclusions are derived in Sect. 4.

## Modelling methodology

Large elastic arteries are characterized by diameters of the order of few centimetres, which decrease in size towards the periphery, where they branch into the smaller muscular arteries (0.1–10 mm in diameter) that perfuse the downstream circulation, called microcirculation. This consists of branching microvessels, called arterioles (below 100 $\upmu$m in diameter), which feed complex capillary networks (Alastruey et al. 2012).

### Equivalent porous media

**Fig. 1:**
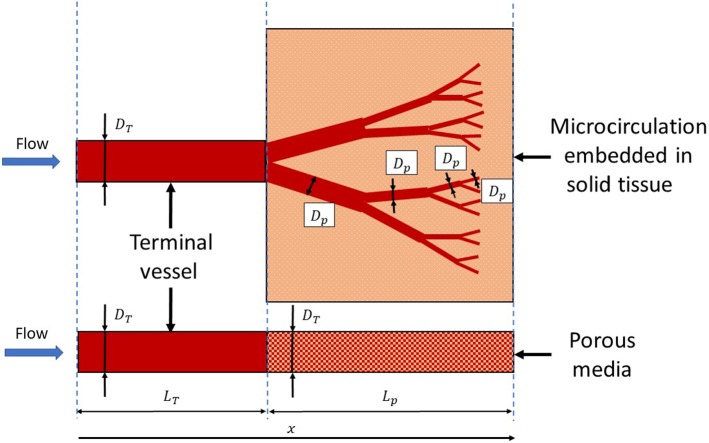
Downstream vascular network embedded in solid tissue (top) and equivalent poro-elastic material model (bottom) for representing terminal boundary conditions

The microcirculatory network, originating from a muscular/small artery, can be seen as series of bifurcations leading to a set of narrower vessels embedded in the solid tissues. The artery where microcirculation branches from is the *terminal vessel* of the arterial systemic circulation considered. The resistance offered by microcirculation to blood flow can be conceived as the one encountered by a fluid when it flows through a porous medium. This means that, for each terminal vessel belonging to the systemic circulation, the downstream vasculature may be represented by a 1D axisymmetric poro-elastic tube (see Fig. 1). For more details about modelling arterial branching and microcirculatory networks, see reference works available in the literature (Pries and Secomb 2008; Pries et al. 2009; Cutri’ et al. 2013; Causin et al. 2016; Arciero et al. 2017).

**Fig. 2:**
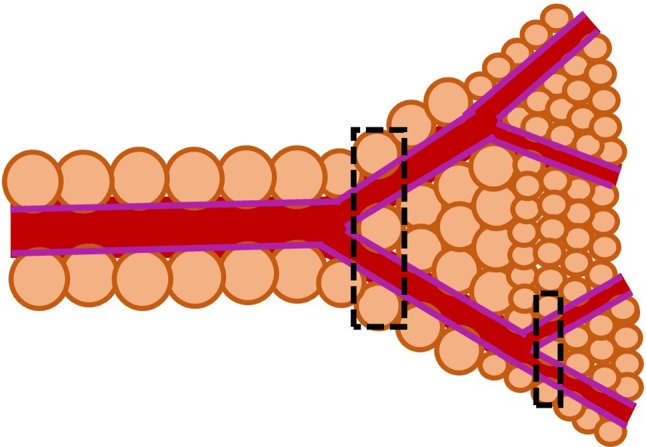
Change of particle size as microvessel diameter decreases. Smaller calibre vessels perfuse more intimately into solid tissue, which can be seen as solid particles embedded in a fluid matrix

By referring to Fig. 1, the characteristics of the proposed porous media model are defined below. The equivalent poro-elastic tube representing the microcirculation in the above figure is assumed to have a variable porosity $\epsilon$ that decreases along the axial coordinate *x*. For a fluid entering into a packed bed, this relationship may be described according to Nithiarasu et al. (1997) and references in this work as:

1 $$\begin{aligned} \epsilon =\epsilon _0 \left[ 1+b_{\mathrm{p}} {\hbox {exp}}\left( -\frac{c_{\mathrm{p}} x}{D_{\mathrm{p}}}\right) \right] , \end{aligned}$$

in which $b_{\mathrm{p}}$ and $c_{\mathrm{p}}$ are empirical constants ($b_{\mathrm{p}}=1.0$, $c_{\mathrm{p}}=2.0$), whilst $\epsilon _0$ is the free stream porosity and $D_{\mathrm{p}}$ is the characteristic dimension, which is taken as the particle size. Since the exponential relationship was developed for packed beds, its applicability to the poro-elastic model of microcirculation may not be very suitable. Thus, we also investigate simpler linear law for describing porosity changes in space, i.e.

2 $$\epsilon = {\left\{ \begin{array}{ll} \epsilon _{\infty } +\frac{(\epsilon _0-\epsilon _{\infty })}{\eta L_{\mathrm{p}}}x &{} 0<x<\eta L_{\mathrm{p}}, \\ \epsilon _0 &{} x \ge \eta L_{\mathrm{p}}, \end{array}\right. } $$

where $\eta$ is a coefficient defining the slope of the variation and $\epsilon _{\infty }$ is the limit porosity. The proposed methodology is based on the assumption that the solid particle diameter is equivalent to the microvessel diameter, as approximately represented in Fig. 2. This modelling hypothesis can be justified for small size vessels, which are intimately embedded in the solid tissue, as reported in Lorthois and Cassot (2010).

For a given porosity $\epsilon$, it is possible to calculate the associated permeability of the medium via (see Nithiarasu et al. 1997)

3 $$\begin{aligned} k_{\mathrm{p}}=\frac{\epsilon ^3 D_{\mathrm{p}}^2}{150 (1-\epsilon )^2}. \end{aligned}$$

The pressure drop associated with the porous media resistance can be obtained through the linear Darcy’s law as

4 $$\begin{aligned} \frac{\partial p_{\mathrm{p}}}{\partial x}=-\mu \frac{u}{k_{\mathrm{p}}}, \end{aligned}$$

where $\mu$ is the fluid viscosity and *u* the fluid velocity averaged over the tube cross-sectional area. The diameter of the poro-elastic tube is set equal to the terminal vessel diameter $D_{\mathrm{T}}$, and $D_{\mathrm{p}}$ values are determined using relationships derived in the following section.

#### Microvascular branching

It is assumed that the microcirculatory network mainly develops along the *x*-axis. All the vessel lengths are considered only in such a direction. The microvessel diameter $D_{\mathrm{p}}$ can be described with a piecewise continuous function, in order to reflect the change in size after each bifurcation. The same can be done for the microvessel length $L_{\mathrm{p}}$. The terminal vessel, having diameter $D_{\mathrm{T}}$ (or $D_{\mathrm{p},0})$ and length $L_{\mathrm{T}}$ (or $L_{\mathrm{p},0}$), branches into two equal narrower and shorter vessels, characterized by a diameter $D_{\mathrm{p},1}$ and length $L_{\mathrm{p},1}$. Each of these vessels branches into other two equal narrower and shorter vessels having diameter $D_{\mathrm{p},2}$ and length $L_{\mathrm{p},2}$, and so on for the following generations.

For a generic vessel generation *n*, characterized by $D_{\mathrm{p},n}$ and $L_{\mathrm{p},n}$, its generation $n+1$ has the size that may be calculated via:

5 $$\begin{aligned} D_{\mathrm{p},n+1}=\phi D_{\mathrm{p},n}\quad {\hbox {and}}\quad L_{\mathrm{p},n+1}=\eta L_{\mathrm{p},n}, \end{aligned}$$

in which the coefficients $\phi$ and $\eta$ are those to ensure that, after each bifurcation, the vessel diameter, length and volume decrease and the vessel total cross-sectional area and lateral surface increase. Following the work by Alastruey (2006), the satisfaction of these conditions requires

6 $$\begin{aligned} \frac{1}{2 \phi }<\eta <\frac{1}{2 \phi ^2}. \end{aligned}$$

From Eq. (5), it is possible to write the size of vessel *n* with respect to the size of the terminal vessel as

7 $$\begin{aligned} D_{\mathrm{p},n}(n,\phi )=\phi ^{n} D_{\mathrm{T}} \quad {\hbox {and}}\quad L_{\mathrm{p},n}(n,\eta )=\eta ^{n} L_{\mathrm{T}}. \end{aligned}$$

A microvascular branching characterized by *n* generations has a total length $L_{\mathrm{B}}=L_{\mathrm{B}}(n)$ (including also the terminal vessel length) that can be expressed as

8 $$\begin{aligned} L_{\mathrm{B}}=\sum _{i=0}^{n} L_{\mathrm{p},i}, \end{aligned}$$

which can also be written as

9 $$\begin{aligned} L_{\mathrm{B}}=\sum _{i=0}^{n} \eta ^{i} L_{\mathrm{T}}=L_{\mathrm{T}}\sum _{i=0}^{n} \eta ^{i}=L_{\mathrm{T}} \bigg ( \frac{1-\eta ^{(n+1)}}{1-\eta } \bigg ). \end{aligned}$$

For a generic cumulative length $L_{\mathrm{B}}$, the associated number of branching generations can be found by re-arranging Eq. (9):

10 $$\begin{aligned} n=\frac{{\hbox {log}}\left[ 1-\frac{L_{\mathrm{B}}}{L_{\mathrm{T}}}(1-\eta )\right] }{{\hbox {log}}(\eta )}-1. \end{aligned}$$

It is noticed that this final formulation is very similar to what was derived in Papageorgiou and Jones (1987). With regard to Fig. 1, the total length of the microvascular network can be written as $L_{\mathrm{B}}=L_{\mathrm{T}}+\sum _{i=1}^{n} L_{\mathrm{p},i}=L_{\mathrm{T}}+L_{\mathrm{p}}$. The diameter $D_{\mathrm{p},n}$ can be expressed as a function of the microcirculation length $L_{\mathrm{p},n}$ and the coefficients $\eta$ and $\phi$:

11 $$\begin{aligned} D_{\mathrm{p},n}(n,\phi )=D_{\mathrm{p},n}(L_{\mathrm{B}},\eta ,\phi ). \end{aligned}$$

This means that by knowing the coefficients $\eta$, $\phi$ and total length of microcirculation network $L_{\mathrm{B}}$, both functions $D_{\mathrm{p},n}$ and $L_{\mathrm{p},n}$ can be defined. It is important to note that, since $n=n(x)$, also $D_{\mathrm{p},n}=D_{\mathrm{p}}(x)$.

An alternative way to define the microcirculatory system is to impose, instead of the length $L_{\mathrm{B}}$ and coefficient $\eta$, the minimum diameter $D_{\mathrm{p,min}}$ that is accounted in the model. In this case the number of generations *n* can be easily found by rearranging the first expression in Eq. (7) as

12 $$\begin{aligned} n=\frac{{\hbox {log}} \left( \frac{D_{\mathrm{p,min}}}{D_{\mathrm{T}}}\right) }{{\hbox {log}} (\phi )}. \end{aligned}$$

According to Alastruey (2006), the coefficient $\eta$ can be computed as

13 $$\begin{aligned} \eta =\frac{n}{n+1}\left( \frac{1}{2 \phi ^2} \right) , \end{aligned}$$

guaranteeing, at the last bifurcation of capillaries, the maximum perfusion with the minimum space occupied by the whole network originated from the terminal vessel. Once $\eta$ is known, it is possible to calculate the length of the whole network through Eq. (8).

### Flow in 1D poro-elastic vessel

The variables considered in a 1D arterial circulation system are the cross-sectional area (*A*), the average values of velocity (*u*), fluid pressure (*p*) and porosity ($\epsilon$) over the cross section. The density ($\rho$) of the fluid and wall is considered constant due to the incompressible nature of the materials assumed. The viscosity ($\mu$) of the fluid is also assumed to be constant. Due to the one-dimensional nature of the model, the shear stress is evaluated using Poiseuille’s flow assumption. For relating pressure and cross-sectional area, a nonlinear relationship used by Formaggia et al. (1999) and Olufsen et al. (2000) is employed. The mass and momentum conservation equations for the flow in a poro-elastic vessel, in terms of area and velocity, are given as,

14 $$\begin{aligned}&\frac{\partial (\epsilon A)}{\partial t}+\frac{\partial (\epsilon Au)}{\partial x}=0, \end{aligned}$$

15 $$\begin{aligned}&\frac{\partial u}{\partial t}+u\frac{\partial u}{\partial x}+\frac{\beta }{2\rho \sqrt{\epsilon A}} \frac{\partial (\epsilon A)}{\partial x}+\frac{\mu u}{\rho } \left( \frac{8\pi }{\epsilon A}+\frac{1}{k_{\mathrm{p}}}\right) =0, \end{aligned}$$

where $\beta$ is a parameter accounting for the wall elasticity. Pressure is assumed to be related to cross-sectional area via

16 $$\begin{aligned} p=p_{\mathrm{ext}}+\beta (\sqrt{\epsilon A}-\sqrt{\epsilon A_0}), \end{aligned}$$

in which $p_{\mathrm{ext}}$ is the transmural pressure and $A_0$ the stress-free vessel area. The conservation equations can also be written in the following compact form

17 $$\begin{aligned} \frac{\partial { \mathbf {U} }}{\partial t}+\frac{\partial { \mathbf {F} }}{\partial x}-{ \mathbf {S} }=0, \end{aligned}$$

where $${ \mathbf {U} }$$ is the primitive variables ($$\epsilon A$$,*u*) vector, and $${ \mathbf {F} }$$ and $${ \mathbf {S} }$$ are, respectively, the convective and source terms. It is important to note that for $$\epsilon $$$$\rightarrow $$ 1, the system reduces to the classical Navier–Stokes equations for flow in elastic vessels.

### Solution method

A brief overview on the finite element solver employed is provided in this section. Equation (17) requires a scheme with a stabilization term to obtain a stable solution. Thus, in this study the locally conservative Taylor Galerkin (LCG) method is used, which is the finite element equivalent of Lax–Wendroff stabilization in finite difference discretization. Using this method, the semi-discrete form of Eq. (17) can be written as,

18 $$\begin{aligned} \frac{{{\Delta \mathbf{{U}}}}}{\Delta t}= & {} -\frac{\partial {\mathbf{{F}}}^n}{\partial x} +{\mathbf{{S}}}^n +\frac{\Delta t^2}{2}\left\{ \frac{\partial }{\partial x}\left[ \mathbf{{H}}^n \left( \frac{\partial {{ \mathbf {F} }}^n}{\partial x}-{\mathbf{{S}}}^n \right) \right] \right. \nonumber \\&\left. - {\mathbf{{Q}}}^n \left( \frac{\partial {\mathbf{{F}}}^n}{\partial x}-{\mathbf{{S}}}^n \right) \right\} , \end{aligned}$$

where ${ \mathbf {H} }$ and $\mathbf{{Q}}$ are, respectively, the Jacobian matrices of the convective and source terms. Applying LCG method, Eq. (18) can be written as (Nithiarasu 2004; Thomas and Nithiarasu 2008):

19 $$\begin{aligned} \begin{aligned} \int _{\Omega _{\mathrm{e}}}{} \mathbf{{N}}^T\,\Delta {\mathbf{{U}}} \hbox {d}x =\,&\Delta t \int _{\Omega _{\mathrm{e}}}{} \mathbf{{N}}^T\,\left( -\frac{\partial {\mathbf{{F}}}^n}{\partial x}+ {\mathbf{{S}}}^n \right) \hbox {d}x \\&+\frac{\Delta t^2}{2} \int _{\Omega _{\mathrm{e}}}{} \mathbf{{N}}^T\,\left\{ \frac{\partial }{\partial x}\left[ \mathbf{{H}}^n\left( \frac{\partial {\mathbf{{F}}}^n}{\partial x}-{\mathbf{{S}}}^n\right) \right] \right. \\&\left. -\, \mathbf{{Q}}^n \left( \frac{\partial {\mathbf{{F}}}^n}{\partial x}- {\mathbf{{S}}}^n\right) \right\} \hbox {d}x, \end{aligned} \end{aligned}$$

The final discrete form of Eq. (19) can now be written as (Mynard and Nithiarasu 2008; Thomas and Nithiarasu 2008)

20 $$\begin{aligned}{}[\mathbf{{M}}_{\mathrm{e}}] \{ \Delta \mathbf{{U}} \}=\Delta t \left( {[\mathbf{{K}_{\mathrm{e}}}] \{ { \mathbf {F} } \}}^n + { [\mathbf{{L}}_{\mathrm{e}}] \{\mathbf{{S}} \}}^n+{{\mathbf{{f}}_{\varGamma _{\mathrm{e}}}}}^n \right) , \end{aligned}$$

where $[\mathbf{{M}}_{\mathrm{e}}]$, $[\mathbf{{K}}_{\mathrm{e}}]$ and $[\mathbf{{L}}_{\mathrm{e}}]$ are the element mass matrix, the coefficient matrix for convection, Taylor–Galerkin and source terms for the coupled continuity and momentum equations, respectively. These element matrices of the system of equations are solved on individual elements, independent of surrounding elements. Information is transmitted between elements via the numerical flux term (${\mathbf{{f}}_{\varGamma _{\mathrm{e}}}}$) that is imposed along the boundaries of each element (Mynard and Nithiarasu 2008; Nithiarasu 2004). The time step restriction of the numerical scheme is

21 $$\begin{aligned} \Delta t= 0.9 \frac{\Delta x_{\mathrm{min}}}{c_{\mathrm{max}}}, \end{aligned}$$

where $\Delta x_{\mathrm{min}}$ is the minimum element size in the finite element mesh and $c_{\mathrm{max}}$ is the maximum wave speed. For further details on dealing with bifurcations and other boundary conditions, refer to Mynard and Nithiarasu (2008).

## Results and discussion

In this section, several problems are studied for validating the proposed methodology and probing the parameter effects on the arterial flow waveforms. In all simulations, the porosity ε for the blood vessel lumens is set as $\epsilon _{\infty }=0.99999$. This will recover the Navier–Stokes equations for flow through elastic vessels with no porous media. After the terminal vessels, the porous resistance is switched on by appropriately imposing varying porosity values as discussed in the previous sections. The coefficient $\phi$ is kept fixed equal to $\sqrt{0.6}$, as suggested in Alastruey (2006).

### Porosity model effect: single vessel

**Fig. 3:**
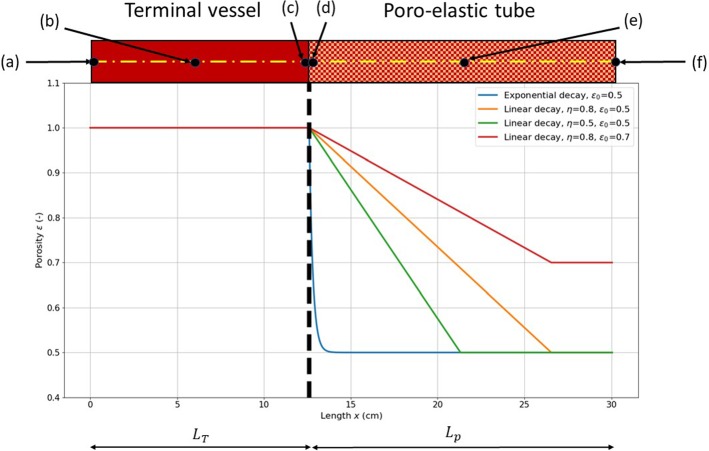
Space–porosity models adopted. The locations (a), (b), (c), (d), (e) and (f) are used to monitor flow rate and pressure

**Fig. 4:**
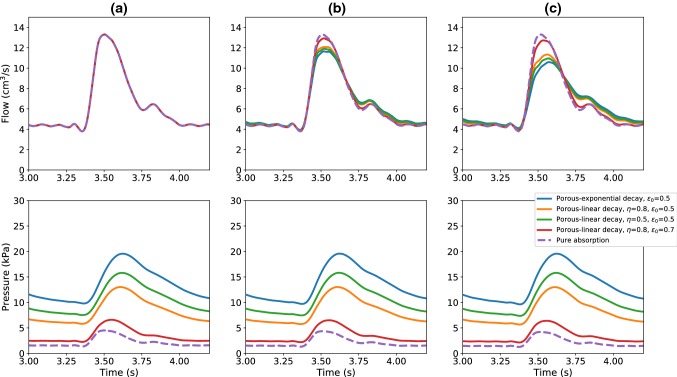
Flow and pressure time evolutions at the monitored locations (**a**), (**b**) and (**c**) of the terminal vessel, for different outflow boundary conditions

In this subsection, the effects of porous media approach used for terminal resistance on flow and pressure are analysed using a single vessel (see Fig. 3). The results are compared with the ones obtained for a vessel with no reflections prescribed at the outlet (pure absorption boundary condition). A vessel representing the carotid artery is considered here. The unstressed vessel cross-sectional area used is $A_0=0.22038$ cm$^2$, the tube length $L_{\mathrm{T}}=12.6$ cm and the elastic parameter $\beta =2251960.3$ dyne/cm$^3$. In addition, the dynamic viscosity $\mu =0.04$ poise and the fluid density $\rho =1.06$ g/cm$^3$ are also used. At the inlet, a flow waveform is prescribed (see Fig. 4). Both the vessel and porous tube domains are discretized with 500 linear finite elements each and the time step used is $\Delta t=0.000005$ s. An analysis of the mesh size effect on the solution is reported in “Appendix”.

For this numerical experiment, four different space–porosity relationships have been used and the minimum microvessel diameter, $D_{\mathrm{p,min}}$, is set equal to 0.2 cm. The length of the porous section $L_{\mathrm{p}}$ is determined using the relationship discussed in Sect. 2.1.1. The space–porosity relationships used are exponential and linear decays with additional parameter variations as shown in Fig. 3. It is important to note that many other porosity variations, including nonlinear variations, can be imposed. Such flexibility allows the model to adapt realistic variations if the porosity variations in real microcirculation can be measured. More details on the measurement of the tissue porosity associated with microvessels can be found in recent studies like Sato et al. (2015), Tang et al. (2015) and Peyrounette et al. (2015). It is worth mentioning that the variations shown in Fig. 3 are only for demonstrating the usability and effectiveness of the proposed model.

Figure 4 shows the time evolution of flow and pressure at monitored locations of the terminal vessel (a), (b) and (c). For the *pure absorption* case both flow and pressure signals are identical at both inlet and exit. As shown in Fig. 4, the employment of the porous media approach results in a magnification of the pressure amplitude. Imposing a variable porosity media, beyond the terminal vessel, increases the resistance the fluid encounters at the terminal vessel outlet. This is reflected by the drop in peak flow at the outlet for all porosity models although the total mass is conserved (see Fig. 4). The exponential decay model is characterized by a much steeper drop in porosity along the length (see Fig. 3), which leads to higher peak pressure than in the linear decay cases as shown in Fig. 4. Among the linearly varying porosity cases, higher pressure is recorded for steeper variation in porosity. This is because steeper porous profiles provide a much higher resistance to flow. As seen, increase in free-porosity parameter $\epsilon _0$ decreases the pressure at the outlet.

**Fig. 5:**
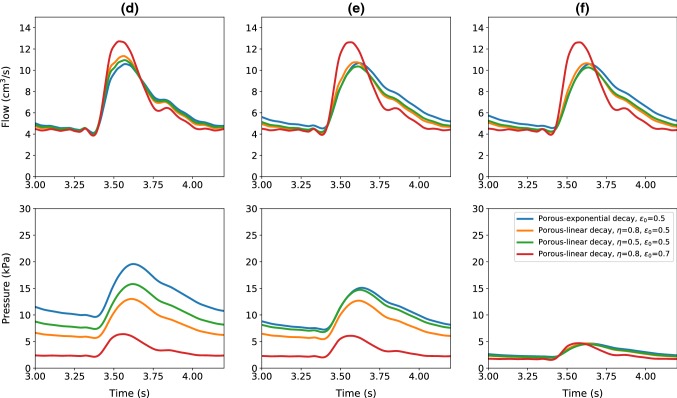
Flow and pressure time evolutions at the monitored locations (**d**), (**e**) and (**f**) of the poro-elastic vessel, for different porosity models

Figure 5 shows the waveforms obtained inside the poro-elastic tube. As seen, the pressure drop increases with distance in a consistence manner in all models employed. This represents a smooth decay in pressure, as normally expected in a microcirculation. Thus, the proposed model, supported by more realistic representation of the porosity variation, can easily replicate microcirculation.

### Microvessel diameter effect: single vessel

In this section the effects of minimum microvessel diameter on flow variables are investigated. Performances of the proposed approach are compared with the classical three elements (resistance–capacitance–resistance, RCR model) Windkessel model. The properties, geometries and boundary conditions for two example problems are taken from Boileau et al. (2015). Once again a single vessel is used but with two different diameters, one representing aorta and another representing carotid artery. The material properties and flow conditions are appropriately changed to reflect the change in nature of the two subproblems. For carotid artery, the conditions are identical to the one explained in the previous subsection. The stress-free area of the aorta is taken as 3.0605 cm$^2$, the elastic parameter $\beta$ is taken equal to 370648.7 dyne/cm$^3$, the length of the aorta used is 24.137 cm, and all other properties remain the same as carotid artery. Each artery is discretized with 500 linear finite elements. The poro-elastic vessel used for applying the boundary condition is also discretized using the same number of elements. Three different minimum microvessel diameters ($D_{\mathrm{p,min}}=0.2, 0.1$ and 0.01 cm) and three space–porosity relationships are considered.

**Fig. 6:**
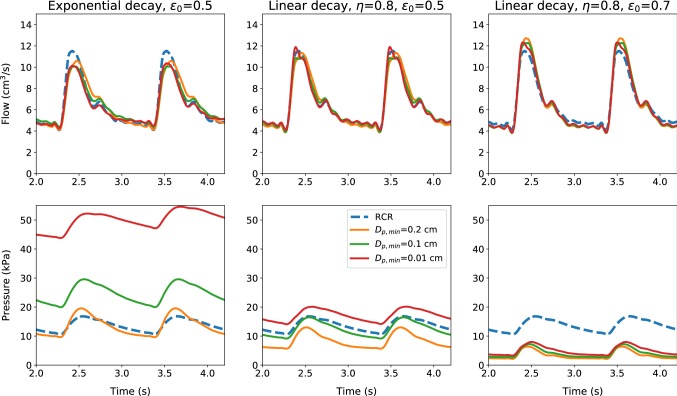
Flow and pressure time evolutions at the outlet of the common carotid artery, for different terminal models and minimum microvessel diameters

Figure 6 shows both flow and pressure evolutions with time at the outlet of the common carotid artery for three porosity-space relationships. This figure also shows the results obtained using the RCR model used in Boileau et al. (2015). By varying the minimum microvessel diameter $D_{\mathrm{p,min}}$, a slight change in flow amplitude is observed. The pressure on the other hand significantly increases with decrease in $D_{\mathrm{p,min}}$. This is anticipated as smaller vessels offer higher resistance and thus increase in pressure. The spatial distribution of the porosity also plays a fundamental role in determining the pressure distribution. The linear decay case with $\eta =0.8$ and $\epsilon _0 =0.5$ provides results that are close to the RCR model when $D_{\mathrm{p,min}}= 0.1$ cm. It appears that both the exponential and linear porosity models can be tuned to match the pressure distribution generated by another model. However, the linear models appear to be giving a better overall control in terms of adapting. Irrespective of these, it would certainly be useful in the future to have the microvessel porosities and diameters measured to represent the resistances more robustly into the porous media models. With regard to the computational efficiency, the poro-elastic model is more expensive than the RCR model (+ 83.1% for $D_{\mathrm{p,min}}= 0.2$ cm), due to the additional equations to be solved for the porous domain. However, this cost is strongly affected by the type of discretization adopted for the porous media. Moreover, it is important to mention that the treatment of the boundary conditions represents a negligible fraction of the overall simulation cost.

**Fig. 7:**
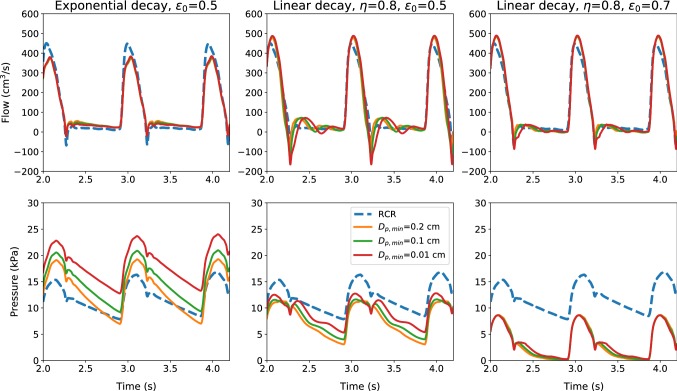
Flow and pressure time evolutions at the outlet of the upper thoracic aorta, for different terminal models and minimum microvessel diameters

In Fig. 7 time evolutions of the flow rate and pressure at the outlet of the upper thoracic aorta are shown. It is important to note that for the exponential porosity decay, the waveform shape is preserved across all $D_{\mathrm{p,min}}$ cases. However, this is not always the case with linear porosity variation. As seen the linear approach not always preserves the waveform shape. This is expected as the proposed porosity models are meant for resistances represented by microcirculation, and thus, their applicability to large vessel resistances may require more work.

### An arterial network

**Fig. 8:**
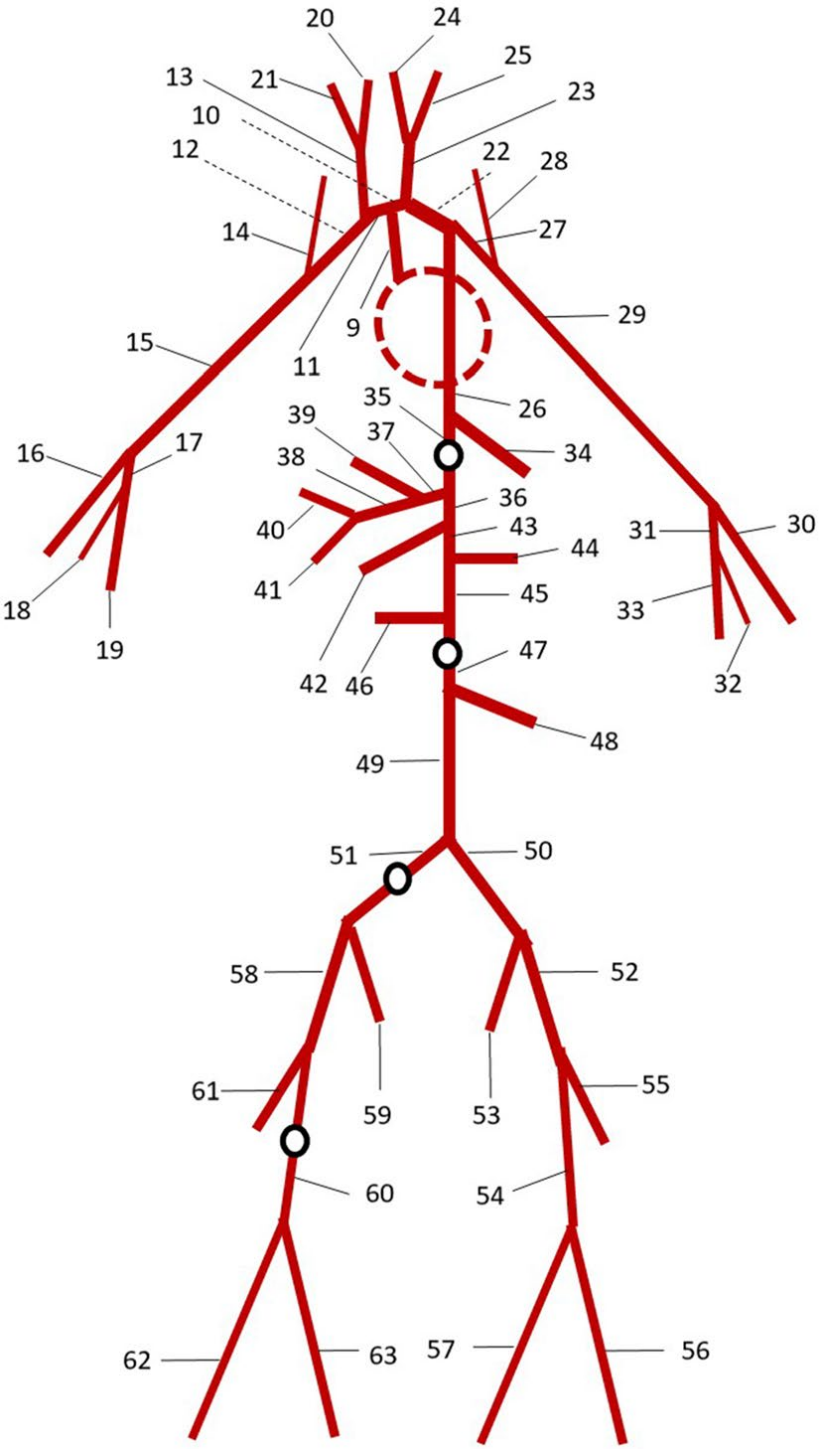
Arterial tree proposed by Low et al. (2012). The red dashed line represents the heart region including the first eight arteries of the network. Black circles indicate the four monitoring points where flow variables are recorded

In this section, the proposed methodology is applied for treating the boundaries in a realistic arterial tree network. The model validation is carried out by studying how waveforms propagate from aorta to the lower limb. The simulation results are compared with in vivo data taken from different studies (Schaaf and Abbrecht 1972; Matthys et al. 2007; Kroeker and Wood 1955; Reymond et al. 2009). The arterial tree adopted is the one described by Low et al. (2012) and is shown in Fig. 8. For more details on the boundary conditions and geometry of the system, refer to the above-cited work. The time step used is $\Delta t=0.00002$ s, and each porous tube beyond the terminal vessel is discretized in space by using an element length of $\Delta x=0.2$ cm. The simulations are carried out for porous media model with different $D_{\mathrm{p,min}}$ (0.08 and 0.05 cm) and two different space–porosity relationships (exponential and linear decays).

Flow variables are monitored at four different locations of the arterial tree. These correspond to the middle of four arteries proposed in Low’s network (empty black circles in Fig. 8): *thoracic aorta II*, *abdominal aorta IV*, *right common iliac* and *right femoral*.

**Fig. 9:**
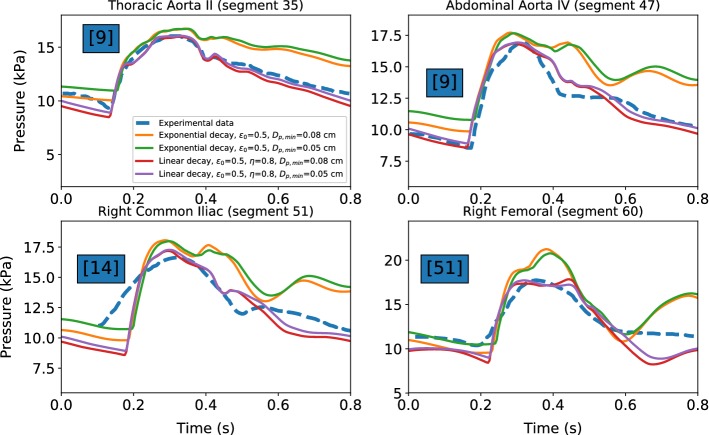
Time evolutions of pressure at different nodes of the arterial network. Dashed lines are used for experimental data from the literature: Schaaf and Abbrecht (1972), Matthys et al. (2007) and Kroeker and Wood (1955)

In Fig. 9, pressure values are reported with respect to time for all four locations. As seen, agreement with the experimental measurements is satisfactory, especially for the model with linear decay in porosity. It also appears that a decrease in minimum diameter of the microvessel slightly increases the accuracy although this is not significant. The exponential decay appears to provide poor approximation, involving an augmentation of the signal period. However, with change in parameters used in the exponential relationship, accuracy could be improved. It is worth noting that the experimental data for the right common iliac artery are taken from a study carried out on an circuit of elastic tubes and not a real arterial system. This may explain the marked discrepancy between experimental and numerical results.

**Fig. 10:**
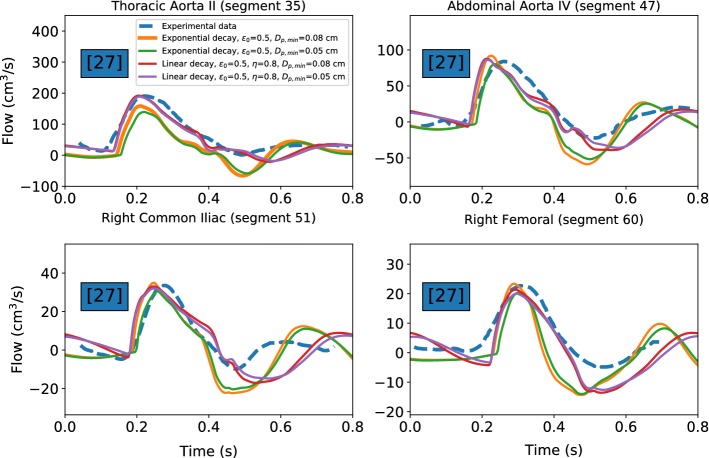
Time evaluations of flow rate at different nodes of the arterial network. Continuous lines are used for simulation results. Dashed lines are used for experimental data from the literature: Reymond et al. (2009)

The time evaluations of flow rate for the mentioned four locations are plotted in Fig. 10. As seen, they in general are in good agreement with the measured data.

## Concluding remarks

In this paper a novel, porous media-based approach is introduced for treating outflow boundary conditions in 1D blood flow modelling. The model’s effects on flow and pressure fields are investigated through a series of numerical examples. The role of parameters like $\epsilon$ and $D_{\mathrm{p,min}}$ is also elucidated. The proposed methodology is then validated against the popular benchmark and network problems. The proposed model has the advantage that no specific regional parameters require to be determined by a tuning process, and only few general parameters, describing the porous media, are user-dependent. The results clearly show that the spatial distribution of the porosity strongly affects the results. The same can be said for the minimum size of the microvessels considered. The simulation results showed that the model is able to provide very similar results to that of the reference results, but the proposed model is robust in the sense that it requires smaller number of parameters. Such parameters can be determined independent of flow. The proposed model can be easily adapted to represent experimental or other model data as it is easy to control. Thus, the proposed model provides a more flexible and simpler alternative to the existing models. Further research is required on experimental measurements of porosity of microcirculation and new porosity variation models.

## Figures and Tables

**Fig. 11:**
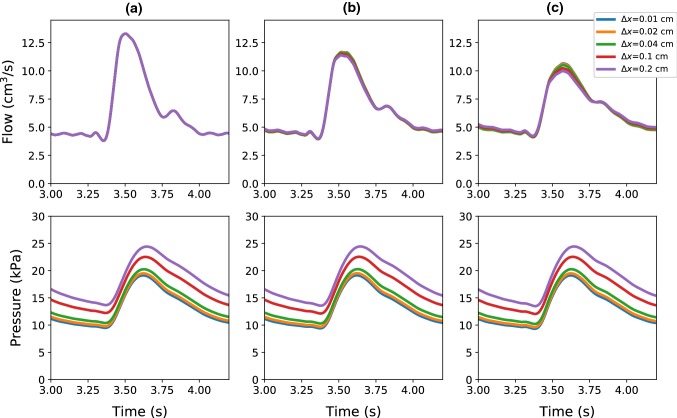
Flow and pressure time evolutions at the monitored locations (**a**, **b**, **c**), for five different mesh sizes

## Appendix: Mesh size effect

In this section the mesh size effect on the solution of the problem in Sect. 3.1 is reported. With regard to the space–porosity relationship, the exponential decay case is considered. In this analysis five different element sizes are considered ($\Delta x=0.01, 0.02, 0.04, 0.1$ and 0.2 cm). It is worth mentioning that the same $\Delta x$ is applied for vessel and porous tube. All the other parameters, including the time step, are the same as in Sect. 3.1. Figure 11 shows flow and pressure values in time at the locations (a), (b) and (c), for five different mesh sizes.

The comparison shows that for mesh size equal and lower than $\Delta x=0.02$ cm the numerical solutions tend to converge to the same values.
